# Plasmacytoid Dendritic Cells Contribute to Systemic but Not Local Antiviral Responses to HSV Infections

**DOI:** 10.1371/journal.ppat.1003728

**Published:** 2013-10-24

**Authors:** Melissa Swiecki, Yaming Wang, Susan Gilfillan, Marco Colonna

**Affiliations:** Department of Pathology and Immunology, Washington University School of Medicine, St Louis, Missouri, United States of America; University of North Carolina at Chapel Hill, United States of America

## Abstract

Plasmacytoid dendritic cells (pDC) produce type I interferons (IFN-I) and proinflammatory cytokines in response to viruses; however, their contribution to antiviral immunity in vivo is unclear. In this study, we investigated the impact of pDC depletion on local and systemic antiviral responses to herpes simplex virus (HSV) infections using CLEC4C-DTR transgenic mice. We found that pDC do not appear to influence viral burden or survival after vaginal HSV-2 infection, nor do they seem to contribute to virus-specific CD8 T cell responses following subcutaneous HSV-1 infection. In contrast, pDC were important for early IFN-I production, proinflammatory cytokine production, NK cell activation and CD8 T cell responses during systemic HSV-2 and HSV-1 infections. Our data also indicate that unlike pDC, TLR3-expressing cells are important for promoting antiviral responses to HSV-1 regardless of the route of virus administration.

## Author Summary

Herpes simplex viruses (HSV) cause a variety of diseases in human from the common cold sore to more severe illnesses such as pneumonia, herpes simplex keratitis, genital herpes and encephalitis. HSV are large double-stranded DNA viruses that infect epithelial or epidermal cells before establishing a latent infection in sensory neurons. Both innate and adaptive immune responses are necessary for limiting viral replication and maintaining latency. Viral detection through distinct pathogen recognition pathways triggers several signaling cascades that lead to the production of proinflammatory cytokines and type I interferons, which establish inflammation, confer an antiviral state and promote immune responses. Our study provides new insights into the cell types and pathogen recognition pathways involved in antiviral defense to HSV at local and systemic barriers and thus, might facilitate the development of novel strategies to treat HSV infections.

## Introduction

Most cells are able to produce type I interferons (IFN-I) in response to viruses, however, some cell types such as plasmacytoid dendritic cells (pDC) are more efficient than others. pDC detect RNA and DNA viruses through two endosomal sensors, Toll-like receptor (TLR) 7 and TLR9, respectively, which induce secretion of IFN-I through the MyD88-IRF7 signaling pathway [1]–[3]. Because of their capacity to produce IFN-I, as well as proinflammatory cytokines, and their ability to present antigens to T cells, pDC are thought to be important for promoting immune responses, particularly to viruses [4], [5].

In order to evaluate the contribution of pDC to innate and adaptive antiviral responses in vivo, depletion studies are warranted. Several mouse models to eliminate pDC have been described. First attempts used antibody (Ab)-mediated depletion of pDC [6]. Within the past few years, genetically modified mouse strains have become available that lack pDC either constitutively [7], [8] or by inducible depletion [9], [10]. CLEC4C-DTR transgenic (Tg) mice have been generated that express the diphtheria toxin receptor (DTR) under the control of the CLEC4C promoter [9]. CLEC4C, also known as blood dendritic cell antigen 2, is a type II C type lectin that is uniquely expressed by human pDC [11], [12]. Injection of diphtheria toxin (DT) into CLEC4C-DTR Tg mice selectively eliminates pDC [9]. Recently, a SiglecH-DTR knockin mouse was described that has an IRES-DTR-EGFP cassette inserted into the SiglecH locus [10]. These mice not only lack SiglecH expression, but can also be depleted of pDC after DT administration. SiglecH is a member of the sialic acid-binding immunoglobulin (Ig)-like lectin family that is routinely used to discriminate pDC from other cell types in mice [13], [14].

Herpes simplex virus (HSV)-1 and HSV-2 are large double-stranded DNA viruses that infect epithelial or epidermal cells before establishing a latent infection in sensory neurons [15]. Both innate and adaptive immune responses are necessary for limiting viral replication and maintaining latency [16]. pDC detect HSV and produce IFN-I and proinflammatory cytokines via TLR9 [17]–[20]. Ab-mediated depletion studies have suggested a critical role for pDC in promoting immunity to HSV both locally and systemically. Because the available pDC-depleting Abs also cross-react with other cell types, we decided to investigate the impact of pDC depletion on local and systemic antiviral responses to HSV infections using CLEC4C-DTR Tg mice. We found that the absence of pDC did not appear to influence antiviral responses to local HSV-2 and HSV-1 infections. In contrast, pDC were important for IFN-I production, NK cell activation and CD8 T cell responses following systemic HSV-2 and HSV-1 infections. Our findings suggest that previous studies highlighting a protective role for pDC during local HSV infections may be related to the depletion of other cell types. Our data also corroborate previously published findings that TLR3-expressing cells, unlike pDC, are critical for antiviral CD8 T cell responses to HSV-1 regardless of the route of administration [21] and are a major source of IFN-I during systemic infection that promotes NK cell activation at later time points post-infection (p.i.). Altogether, our study demonstrates that the cellular sources of IFN-I and antigen-presenting cells that mediate antiviral responses to HSV infections vary depending on the route of infection.

## Results

### Limited impact of pDC on viral burden, local IFN-I and survival during vaginal HSV-2 infection

**Figure 1:**
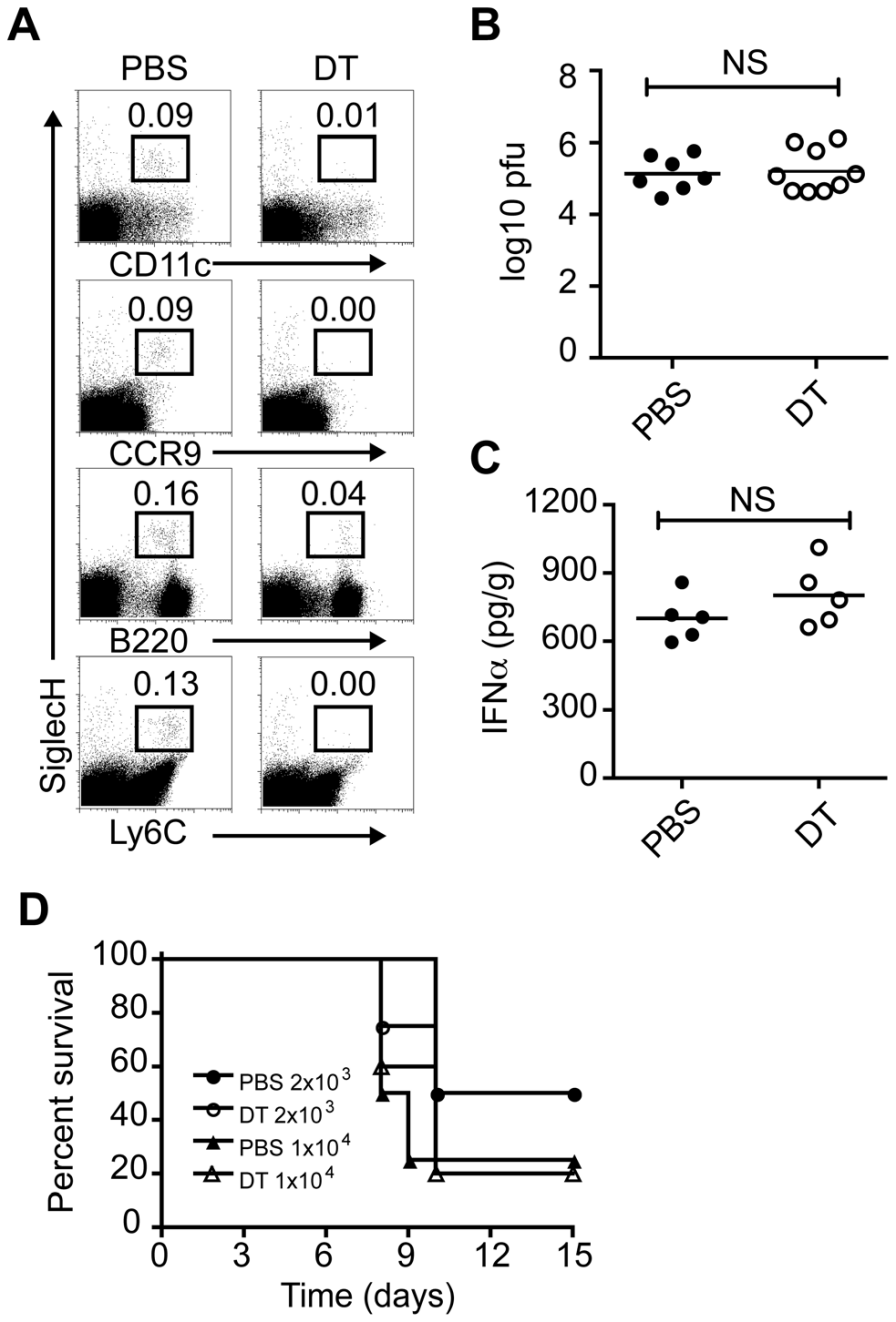
Impact of pDC on vaginal HSV-2 infection. CLEC4C-DTR Tg mice were injected subcutaneously with Depo-Provera or medroxyprogesterone acetate 7 days before vaginal HSV-2 infection. Mice were injected i.p. with PBS or DT 24 h before infection and every other day after. (A) Dotplots show pDC frequencies in lumbar lymph nodes of PBS or DT-treated mice at the time of infection. HSV-2 titers (B) and IFN-α levels (C) in vaginal/cervical tissues on day 2 p.i. after inoculation with 1×10^3^ pfu. (D) Control or pDC-depleted mice were infected with 2×10^3^ or 1×10^4^ pfu of HSV-2 and survival was monitored for 15 days. Data are combined from or are representative of two independent experiments. Not significant (NS).

It has been reported that in humans pDC infiltrate the dermis of recurrent genital herpes simplex lesions and are closely associated with T cells and NK cells and thus, may contribute to control of recurrent herpes virus infection in vivo [22]. pDC have also been detected in the vaginal mucosa of mice infected with HSV-2 [23], [24]. Ab-mediated depletion of pDC using 120G8 [25] or mPDCA1 [26] during vaginal HSV-2 infection impairs innate antiviral responses [23]. pDC-depleted mice showed higher viral titers on days 2 and 3 p.i., reduced IFN-I responses and survival following HSV-2 challenge [23]. Since the available antibodies to deplete pDC, which include 120G8 [25], mPDCA1 [26], 927 [27] and Gr-1 [28], [29], also react with other cell types we decided to evaluate the involvement of pDC in vaginal HSV-2 infection using genetically modified mice where pDC are selectively eliminated after DT administration [9]. To this end, CLEC4C-DTR Tg mice were injected with phosphate buffered saline (PBS) or DT then infected intravaginally (ivag) with HSV-2. We found that pDC in the lumbar lymph nodes, which drain the vagina, were efficiently depleted at the time of infection (Figure 1A). Depletion of pDC did not impact viral burden or IFN-α in vaginal tissues on day 2 p.i. (Figure 1B and 1C). We next determined whether pDC ablation impaired survival to HSV-2 infection. Mice were depleted of pDC or not and challenged with two different doses of HSV-2 ivag (Figure 1D). Survival was monitored for 15 days. Both groups of mice infected with 1×10^4^ pfu of virus had a survival rate of approximately 20% by day 15. When a lower dose of virus was administered (2×10^3^ pfu), around 50% of control and pDC-depleted mice succumbed to infection by day 15. These results suggest that pDC do not strongly influence viral burden or survival following vaginal HSV-2 infection and that previous findings describing a major role for pDC in antiviral responses to HSV-2 may be related to the depletion of other cell types that are critical for controlling vaginal HSV-2 infection.

### pDC do not contribute to antiviral CD8 T cell responses following subcutaneous HSV-1 infection

**Figure 2:**
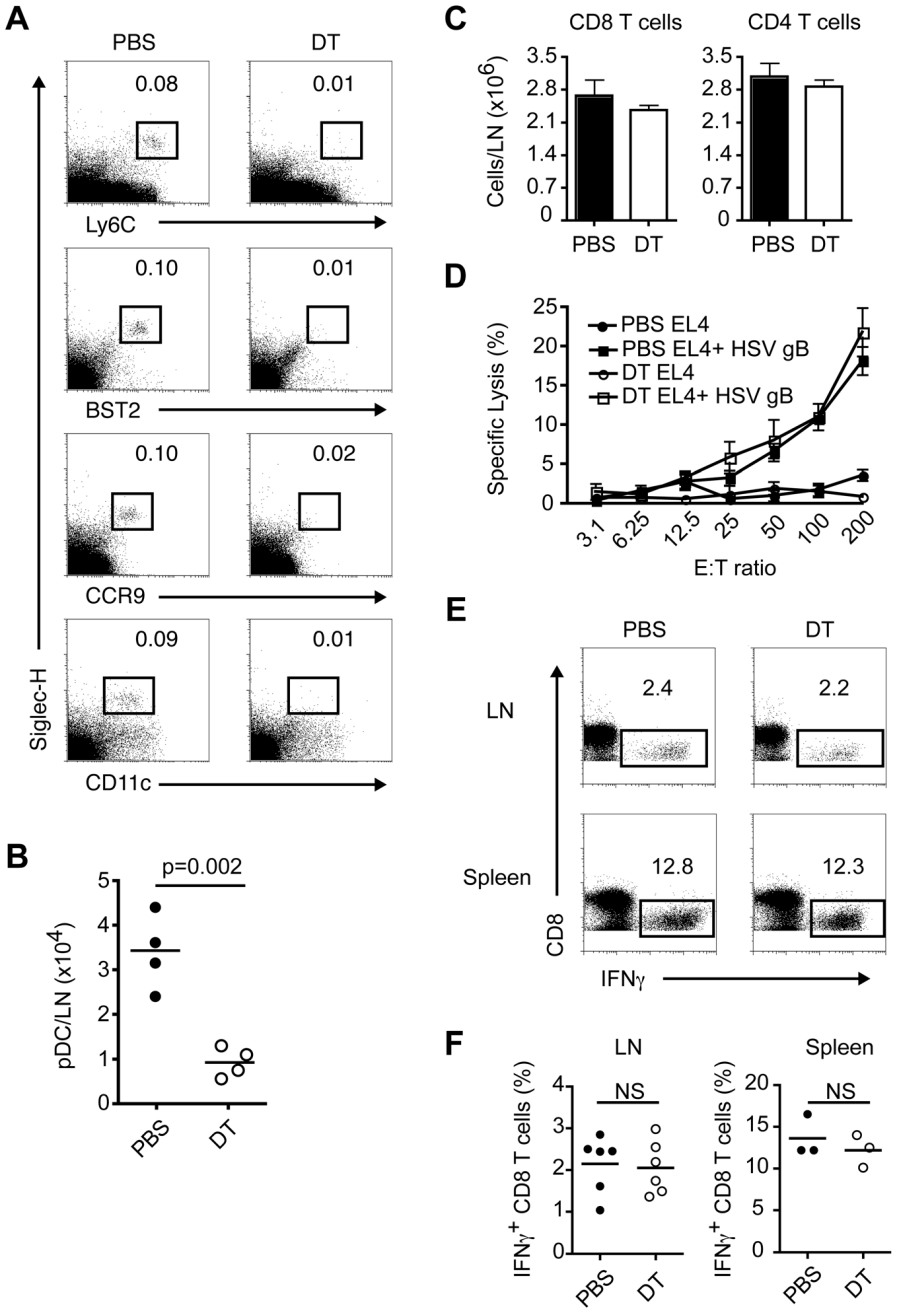
Contribution of pDC to virus-specific CD8 T cell responses after local HSV-1 infection. Control (PBS) and pDC-depleted (DT) mice were infected in the footpad with HSV-1. Draining popliteal lymph nodes (DLN) and spleens were analyzed on day 7 p.i. (A) Dotplots show pDC frequencies in popliteal lymph nodes of PBS or DT-treated mice at the time of infection. (B) pDC numbers in DLN of PBS or DT-treated mice on day 7 p.i. (C) Total numbers of CD8 and CD4 T cells in DLN. (D) Antigen-specific lysis using DLN cells as effectors and HSV gB peptide-pulsed or unpulsed EL4 cells as targets was assessed in standard 4 h ^51^Cr release assays. (E, F) Frequencies of CD8 T cells producing IFN-γ in DLN and spleens after restimulation with HSV gB peptide. Data are combined from or are representative of two independent experiments.

It has also been described, using Ab-mediated ablation, that pDC are imperative for optimal HSV-1-specific CD8 T cell responses after cutaneous infection [30]. In this study, pDC depletion impaired DC functions and cytotoxic T cell-mediated virus eradication in draining lymph nodes (DLN) [30]. Given that Ab administration may potentially deplete non-pDC in this model as well, we decided to evaluate local anti-HSV-1 CD8 T cell responses in CLEC4C-DTR Tg mice depleted or not of pDC. First, we confirmed that pDC are efficiently depleted in the popliteal lymph nodes of DT-treated mice (Figure 2A). We then infected control and depleted mice with HSV-1 in the footpad. On day 7 p.i. we measured total numbers of pDC, CD8 and CD4 T cells in DLN; the ability of cells from DLN to lyse target cells pulsed with HSV gB peptide; frequencies of gB-specific CD8 T cells in DLN and spleen; and viral burden in DLN (Figure 2 and data not shown). Our results show that pDC were largely reduced in the DLN of DT-treated mice on day 7 p.i. suggesting their absence throughout the course of infection (Figure 2B) and that absolute numbers of CD8 and CD4 T cells in DLN were comparable between PBS and DT-treated mice (Figure 2C). We also found using standard chromium release assays that cells from DLN of PBS and DT-treated mice were equally capable of lysing peptide-pulsed target cells (Figure 2D). Furthermore, when cells from DLN and spleen were restimulated with HSV gB peptide both PBS and DT-treated mice had approximately 2% of total CD8 T cells in DLN and 12% of total CD8 T cells in spleen that produced IFN-γ by intracellular staining (Figure 2E and 2F). Finally, we attempted to measure viral burden in DLN by plaque assay on day 7 p.i. but were not able to detect HSV-1 in either PBS or DT-treated mice (data not shown). This is in contrast to the study by Yoneyama *et al.* which detected HSV-1 in DLN on day 7 p.i. in pDC-depleted mice [30]. Taken together these data suggest that the contribution of pDC to antiviral CD8 T cell responses during local HSV-1 infection is negligible in the CLEC4C-DTR Tg mouse model.

### pDC produce cytokines and promote NK cell activation in response to systemic HSV-2 infection

**Figure 3:**
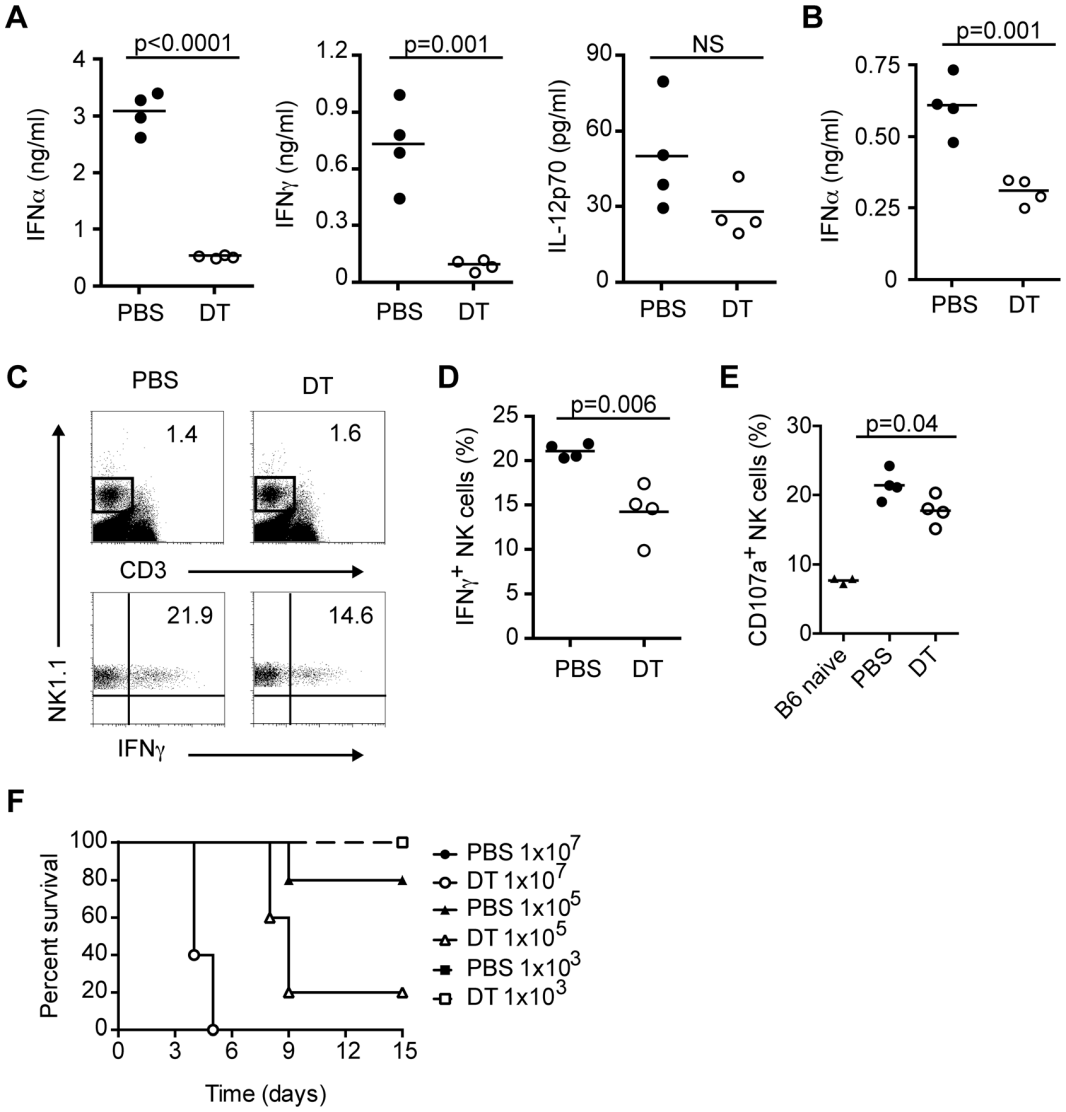
pDC produce IFN-I and activate NK cells during systemic HSV-2 infection. CLEC4C-DTR Tg mice were depleted (DT) or not (PBS) of pDC then infected i.v. with HSV-2 (5×10^6^ pfu). (A) Serum IFN-α, IFN-γ and IL-12p70 levels were measured 8 h p.i. (B) Serum IFN-α levels at 12 h p.i. in control and pDC-depleted mice. Frequencies of IFN-γ-producing NK cells (NK1.1^+^CD3^−^) (C, D) and CD107a^+^ NK cells (E) in spleens were determined 12 h p.i. (F) PBS and DT-treated mice were infected i.v. with different doses of HSV-2. Survival was monitored for 15 days. Data are representative of two independent experiments. Statistical significance is indicated by *p* values. Not significant (NS).

**Figure 4:**
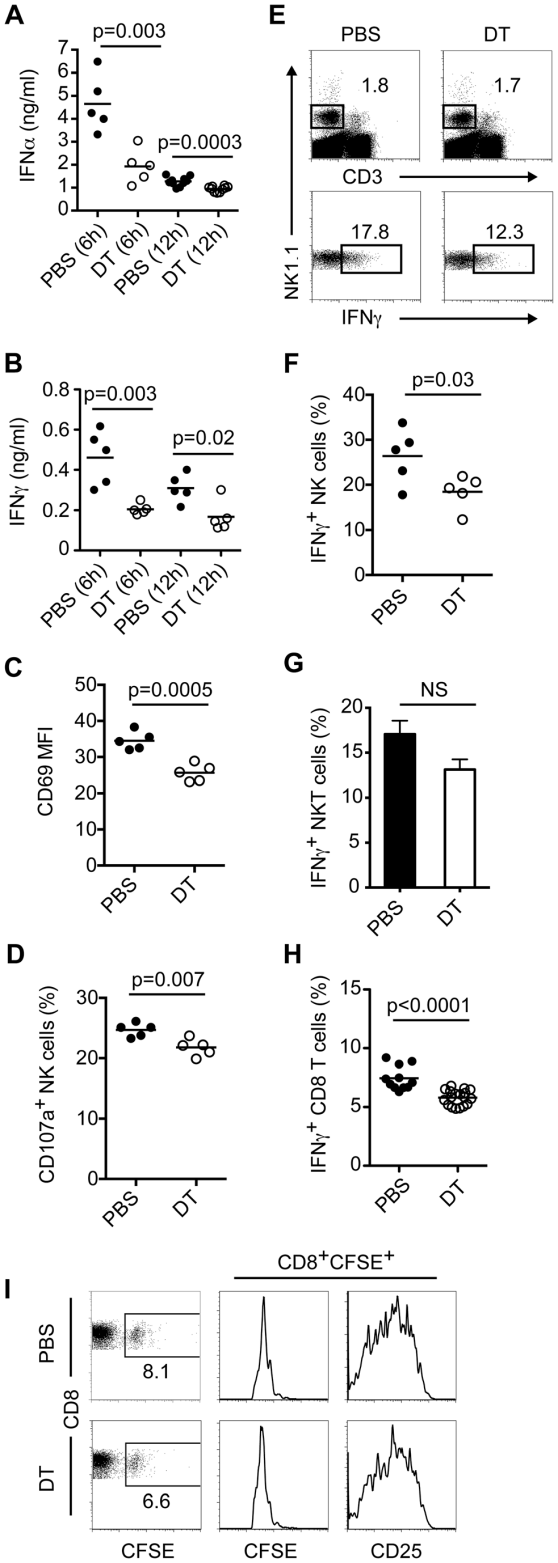
pDC promote antiviral responses during systemic HSV-1 infection. Control (PBS) and pDC-depleted (DT) mice were infected i.v. with HSV-1 (1×10^7^ pfu). Serum IFN-α (A) and IFN-γ (B) were measured at 6 and 12 h p.i. (C) CD69 expression on NK cells (NK1.1^+^CD3^−^) from spleens of PBS or DT-treated mice 12 h p.i. Frequencies of CD107a^+^ (D) and IFN-γ-producing NK cells (E, F) in spleens of HSV-1 infected mice 12 h p.i. (G) Frequencies of IFN-γ-producing NKT cells (NK1.1^+^CD3^+^) in spleens of HSV-1 infected mice 12 h p.i. (H) Frequencies of IFN-γ-producing CD8 T cells in spleens on day 7 p.i. after restimulation with HSV gB peptide. (I) CFSE-labeled CD8 T cells from gBT-I mice were injected i.v. into CLEC4C-DTR Tg mice. Mice were depleted or not of pDC and infected i.v. with HSV-1 (1×10^7^ pfu). Spleens were analyzed on day 3 p.i. for numbers of transferred CD8 T cells, CFSE dilution and CD25 levels. Dotplots and histograms are representative of four mice per group. Data are representative of one (I) or two-three independent experiments (A–H). Statistical significance is indicated by *p* values. Not significant (NS).

Previous studies using either Ab-mediated depletion of pDC or SiglecH-DTR knockin mice injected with DT to eliminate pDC have demonstrated a major role for pDC in innate and/or adaptive responses to systemic HSV-2 or HSV-1 infections [10], [31], [32]. Depletion of pDC with Ab in mice infected systemically with HSV-2 results in reduced serum IFN-I levels, augmented IL-17 production and higher viral burden in liver [31], [32]. In the case of HSV-1, SiglecH-DTR knockin mice depleted of pDC have impaired systemic IFN-α levels and virus-specific CD8 T cell responses [10]. Therefore, we next decided to investigate whether pDC depletion in our mouse model impacted antiviral responses to systemic HSV infections. To this end, we injected CLEC4C-DTR Tg mice with PBS or DT then infected mice intravenously (i.v.) with either HSV-2 (Figure 3) or HSV-1 (Figure 4). In the absence of pDC, there was a dramatic reduction in serum IFN-α and IFN-γ levels 8 h after systemic HSV-2 infection (Figure 3A). IL-12p70 levels also appeared to be reduced in pDC-depleted mice but numbers did not reach statistical significance (Figure 3A). Serum IFN-α levels were still decreased in pDC-depleted mice at 12 h p.i. (Figure 3B) and NK cell activation, as measured by intracellular IFN-γ and CD107a staining, was impaired in the absence of pDC (Figure 3C–3E). However, in contrast to the study by Stout-Delgado *et al.* [32], serum IL-17 levels were undetectable in our PBS or DT-treated mice (data not shown). Finally, we performed a dose response experiment to determine whether pDC-depleted mice were more susceptible to systemic HSV-2 infection (Figure 3F). Mice were injected with PBS or DT and infected with different doses of HSV-2 i.v. At a dose of 1×10^7^, mice in both groups died on days 4 and 5, while at the lowest dose (1×10^3^) all mice survived. Infection with 1×10^5^ pfu per mouse revealed a strong difference in survival between control and pDC-depleted mice (80% versus 20%). Taken together, these data indicate that pDC promote antiviral responses and play a protective role during systemic HSV-2 infection.

### pDC are critical for optimal antiviral responses to systemic HSV-1 infection

Similar to our findings with systemic HSV-2 infection, CLEC4C-DTR Tg mice depleted of pDC had impaired antiviral responses to systemic HSV-1 (Figure 4). Analyses of serum IFN-α (Figure 4A) and IFN-γ (Figure 4B) levels revealed that pDC-depleted mice had lower amounts of both cytokines compared to PBS-treated mice at 6 h p.i. Although pDC-depleted mice had ∼3 fold less serum IFN-γ at 12 h p.i. (Figure 4B), they had a modest reduction in levels of IFN-α (Figure 4A) compared to control mice, suggesting that other cell types and/or viral sensors are involved in sensing systemic HSV-1 infection and producing IFN-I. Examination of spleens from HSV-1-infected mice at 12 h p.i. revealed that NK cells from pDC-depleted mice expressed lower levels of CD69 (Figure 4C). Additionally, spleens from DT-treated mice had lower frequencies of CD107a^+^ and IFN-γ-producing NK cells compared to PBS-treated mice (Figure 4D–4F). We also evaluated whether CD4 T cells and NKT cells produced IFN-γ in infected control and pDC-depleted mice (data not shown and Figure 4G). Although we did not detect IFN-γ production by CD4 T cells at this time point, we did observe similar frequencies of IFN-γ-producing NKT cells from PBS and DT-treated mice.

On day 7 p.i. we measured virus-specific CD8 T cell responses in spleens by intracellular IFN-γ staining after restimulation with HSV gB peptide (Figure 4H). pDC-depleted mice showed a 25–30% reduction in IFN-γ-producing CD8 T cells compared to PBS-treated mice. We have previously shown that pDC do not prime virus-specific CD8 T cells but appear to promote their survival during vesicular stomatitis virus infection [9]. To determine how pDC impact virus-specific CD8 T cells during systemic HSV-1 infection we injected CFSE-labeled CD8 T cells from gBT-I Tg mice [33] into CLEC4C-DTR Tg mice. Following administration of PBS or DT and infection with HSV-1 we measured accumulation and proliferation of gB-specific CD8 T cells in spleens on day 3 p.i. (Figure 4I). Transferred T cells accumulated in control and pDC-depleted mice and exhibited similar levels of CFSE dilution and CD25, suggesting that the absence of pDC may alter the functional ability of virus-specific CD8 T cells to produce IFN-γ rather than their proliferation or accumulation.

Although our IFN-I and CD8 T cell results during systemic HSV-1 infection are similar to those obtained using pDC-depleted SiglecH-DTR knockin mice [10], we were not able to detect viral replication in spleens of either PBS or DT-treated CLEC4C-DTR Tg mice on day 7 p.i. (data not shown) in contrast to the Takagi *et al.* study. This discrepancy may reflect differences in the strains of HSV-1 used (KOS versus strain F) or differences in the promoter used to drive DTR expression (CLEC4C versus SiglecH). It has been shown that SiglecH is expressed by specialized macrophage subsets and progenitors of pDC and classical DC [14], [34], [35]. Thus, it is possible that certain SiglecH^+^ subsets other than pDC are functionally altered and/or depleted in SiglecH-DTR knockin mice thus yielding stronger phenotypes (i.e. major reductions in IFN-I and/or CD8 T cell responses and increased viral replication) than what we observe in CLEC4C-DTR Tg mice.

### TLR3-expressing cells contribute to antiviral responses after systemic and local HSV-1 infections

**Figure 5:**
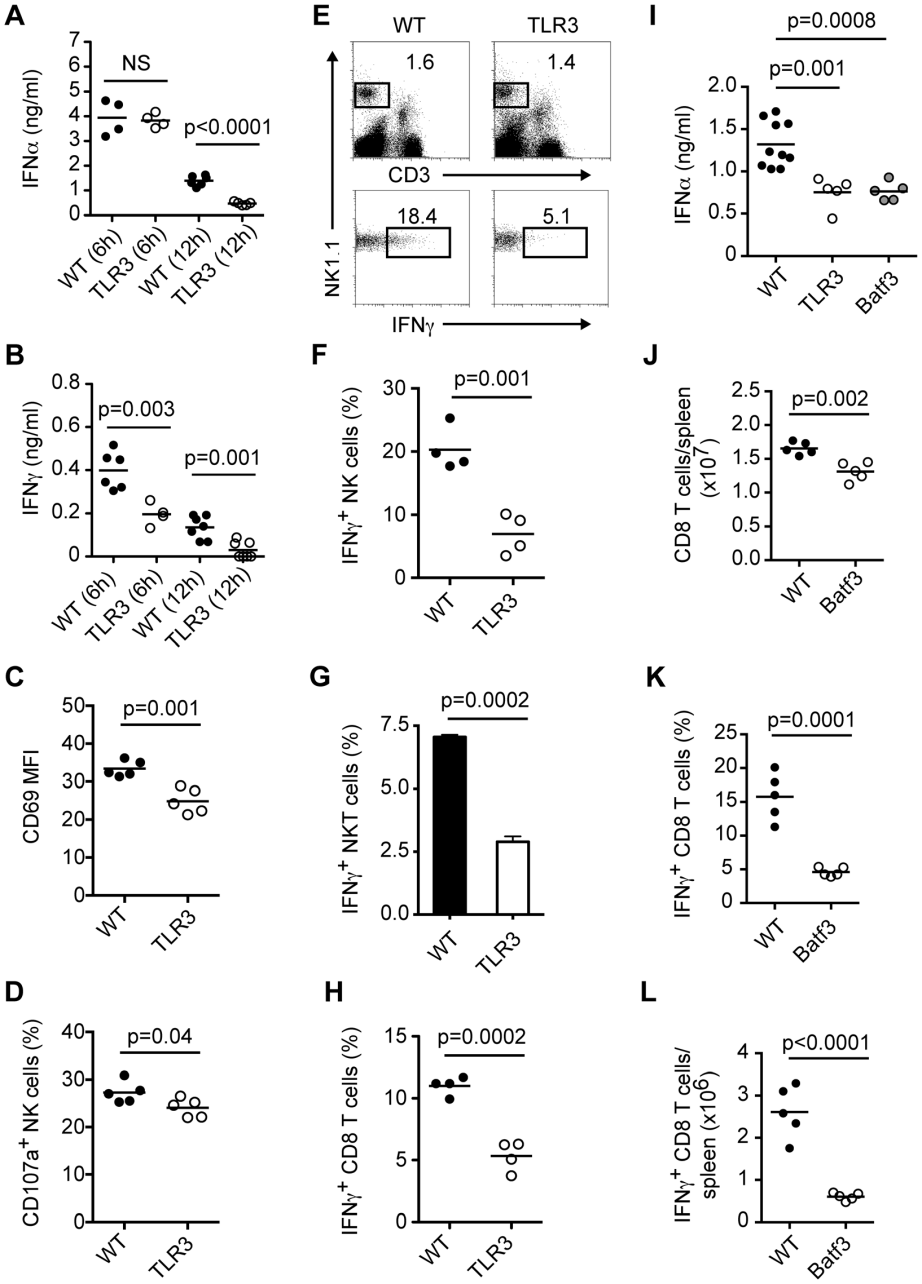
TLR3-expressing cells are necessary for robust antiviral responses during systemic HSV-1 infection. (A–H) WT and TLR3^−/−^ mice were infected i.v. with HSV-1 (1×10^7^ pfu). Serum IFN-α (A) and IFN-γ (B) were measured at 6 and 12 h p.i. (C) CD69 expression on NK cells (NK1.1^+^CD3^−^) from spleens of WT and TLR3^−/−^ mice 12 h p.i. Frequencies of CD107a^+^ (D) and IFN-γ-producing NK cells (E, F) in spleens of HSV-1 infected mice 12 h p.i. (G) Frequencies of IFN-γ-producing NKT cells (NK1.1^+^CD3^+^) in spleens of HSV-1 infected mice 12 h p.i. (H) Frequencies of IFN-γ-producing CD8 T cells in spleens on day 7 p.i. after restimulation with HSV gB peptide. (I) WT, TLR3^−/−^ and Batf3^−/−^ mice were infected i.v. with HSV-1 (1×10^7^ pfu). Serum IFN-α levels were measured 12 h p.i. (J–L) WT and Batf3^−/−^ mice were infected i.v. with HSV-1 (1×10^7^ pfu). (J) Total CD8 T cells in spleens on day 7 p.i. Frequencies (K) and numbers (L) of IFN-γ-producing CD8 T cells in spleens on day 7 p.i. after restimulation with HSV gB peptide. Data are representative of two or three independent experiments. Statistical significance is indicated by *p* values. Not significant (NS).

Although we detected reduced IFN-α responses in pDC-depleted mice 6 h post-HSV-1 infection, at 12 h p.i. there were modest differences in IFN-α levels between control and pDC-depleted mice (Figure 4A). These data indicate that pDC are not the only source of IFN-α during systemic HSV-1 infection and prompted us to examine which sensor was responsible for IFN-α production in the presence or absence of pDC at later time points. TLR3 has a prominent role in HSV-1 recognition and IFN-I production [36]–[38]. In human, TLR3 has proven essential for the recognition of HSV-1 in the brain most likely though the generation of RNA intermediates that occur during viral replication that gain access to the endosomal compartment by phagocytosis or endocytosis [36]–[39]. Thus, we infected wildtype (WT) and TLR3^−/−^ mice i.v. with HSV-1 and measured serum IFN-α levels at 6 and 12 h p.i. We found that at 6 h p.i. TLR3-deficiency did not impact IFN-α levels; however, at 12 h p.i. IFN-α production was greatly diminished in TLR3^−/−^ mice (Figure 5A). These data indicate that pDC are important for the early burst of IFN-α during HSV-1 infection while TLR3 signaling is essential for the later burst of IFN-α.

We also found that serum IFN-γ levels were decreased in TLR3^−/−^ mice at 6 and 12 h p.i. (Figure 5B), suggesting a defect in NK cell activation. Examination of spleens at 12 h p.i. revealed that NK cells from TLR3^−/−^ mice expressed lower levels of CD69 (Figure 5C). Moreover, spleens from TLR3^−/−^ mice had reduced numbers of CD107a^+^ and IFN-γ-producing NK cells compared to infected WT mice (Figure 5D–5F). We also looked at IFN-γ production by CD4 T cells and NKT cells in WT and TLR3^−/−^ mice 12 h p.i. and observed a reduction in activated NKT cells but not CD4 T cells (Figure 5G and data not shown). Prior work has demonstrated that in mice TLR3 expression and CD8α DC, which express high levels of TLR3, are important for virus-specific CD8 T responses following subcutaneous, intravenous and flank HSV-1 infections [21], [40]. To this end, we infected WT and TLR3^−/−^ mice i.v. with HSV-1 and measured virus-specific CD8 T cells in spleens on day 7 p.i. by intracellular IFN-γ staining after restimulation with HSV gB peptide (Figure 5H). TLR3^−/−^ mice showed a ∼50% reduction in IFN-γ-producing CD8 T cells compared to WT mice. Next, we measured serum IFN-α levels and CD8 T cell responses in Batf3^−/−^ mice, which have a defect in CD8α DC numbers [41], [42], after systemic HSV-1 infection. Interestingly, Batf3^−/−^ mice produced lower levels of IFN-α compared to WT mice 12 h p.i., which were similar to those observed in TLR3^−/−^ mice (Figure 5I). Analyses of spleens on day 7 p.i. revealed that Batf3^−/−^ mice had fewer numbers of total CD8 T cells (Figure 5J) and had reduced frequencies and numbers of IFN-γ-producing CD8 T cells after restimulation with HSV gB peptide (Figure 5K and 5L). Thus, we conclude that while pDC contribute modestly to late IFN-α production and CD8 T cell responses during systemic HSV-1 infection, TLR3-expressing cells, most likely CD8α DC, are required for late IFN-α production and efficient priming and/or expansion of virus-specific CD8 T cells [21], [40], [42].

**Figure 6:**
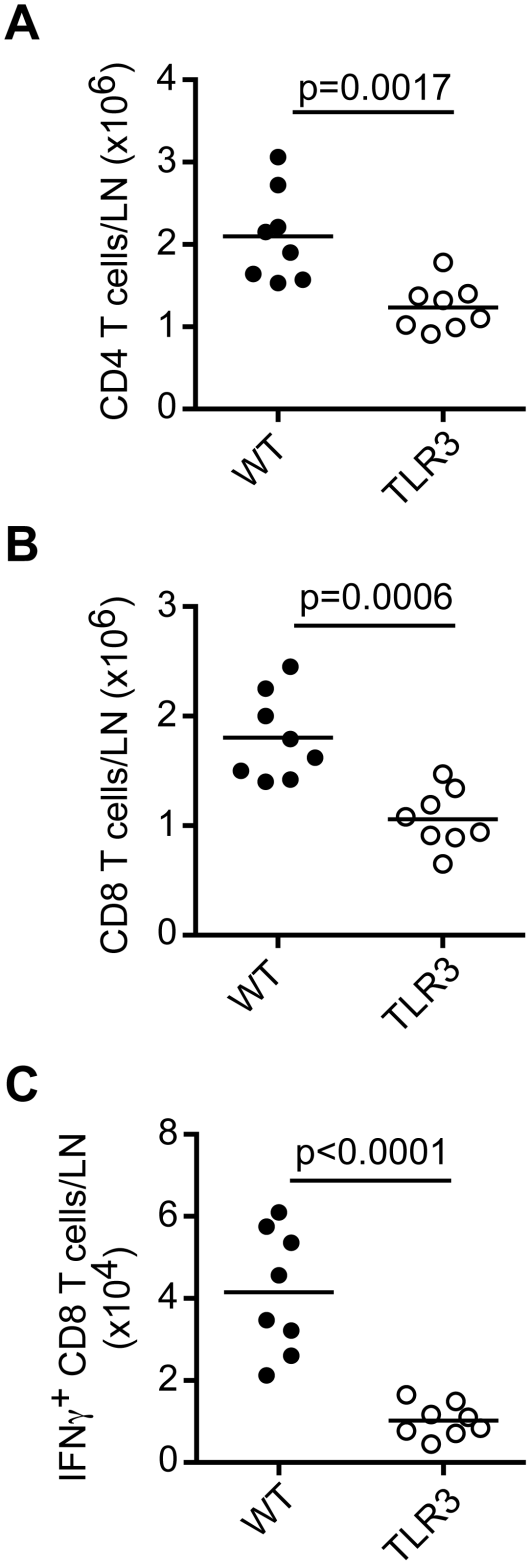
TLR3-expressing cells promote CD8 T cell responses to local HSV-1 infection. WT and TLR3^−/−^ mice were infected in the footpad with HSV-1 (1×10^5^ pfu). Total CD4 (A) and CD8 (B) T cells were analyzed in DLN on day 7 p.i. (C) Numbers of IFN-γ-producing CD8 T cells in DLN on day 7 p.i. after restimulation with HSV gB peptide. Data are combined from two independent experiments. Statistical significance is indicated by *p* values.

Finally, to confirm that TLR3 expression is critical for antiviral responses to HSV-1 regardless of the route of infection we examined T cell numbers and responses in DLN of TLR3^−/−^ mice following footpad infection. DLN from TLR3^−/−^ mice contained fewer numbers of total CD4 and CD8 T cells (Figure 6A and 6B) as well as reduced numbers of IFN-γ-producing CD8 T cells after restimulation with HSV gB peptide (Figure 6C). Taken together, these data corroborate previous findings and illustrate the importance of TLR3 expression in antiviral responses to HSV-1.

## Discussion

In this study, we evaluated antiviral responses during local and systemic HSV infections in the presence and absence of pDC using CLEC4C-DTR Tg mice. We found that pDC have a negligible impact on viral burden, local IFN-I production and survival during vaginal HSV-2 infection and virus-specific CD8 T cell responses after subcutaneous HSV-1 infection. In contrast, pDC were essential for optimal IFN-I production, NK cell activation, CD8 T cell responses and survival following systemic HSV-2 and HSV-1 infections. These findings suggest that the contribution of pDC to antiviral immunity is dependent on the route of virus entry. Although pDC are often recruited to sites of infection and inflammation [43], [44], in homeostatic conditions they are mainly found in circulation and lymphoid tissues, which may explain why they have a more prominent role in controlling systemic viral infections. Indeed, it has been reported that following local infections with viruses such as Newcastle disease virus and vesicular stomatitis virus, macrophages are major sources of IFN-I and promote antiviral responses [45], [46].

Previous studies concluding a major role for pDC in controlling local viral infections, such as HSV, may have been a consequence of depleting other cell types in addition to pDC. This is a likely scenario given that the currently available antibodies to deplete pDC cross-react with other cell types. In early studies, pDC were depleted with a monoclonal antibody (mAb) specific for the antigen Gr-1 [28], [29], which is shared by Ly6C and Ly6G. Ly6C is expressed on pDC, but also on plasma cells, activated T cells and inflammatory monocytes while Ly6G is expressed on granulocytes [47]–[49]. Several other pDC-depleting mAbs have been developed, including 120G8, mPDCA1 and 927 [25]–[27]. These mAbs recognize bone marrow stromal cell Ag 2 (BST2). Although BST2 is selectively expressed on mouse pDC in naïve mice, it is also expressed by plasma cells and is upregulated on most cell types, including classical DC, B cells, T cells, myeloid cells, NKT cells and stromal cells following viral infections or stimulation with IFNs [27]. Therefore, it is important to corroborate these conclusions in mice that specifically lack pDC either transiently [9], [10], constitutively [7], [8] or in mice that have a functional defect of pDC [50], [51].

In contrast to pDC-deficient mice, TLR3^−/−^ mice display impaired antiviral responses to HSV-1 regardless of the route of virus entry. It has previously been reported that TLR3 is required for the generation of CD8 T cell immunity to HSV-1 after flank infection [21] and our results indicate that TLR3^−/−^ mice have reduced virus-specific CD8 T cell responses after systemic and footpad infections. CD8α DC, which express high levels of TLR3, are essential for inducing T cell-mediated immunity to HSV following subcutaneous and systemic infections [40], [42]. The significance of TLR3 in sensing HSV has been corroborated by studies showing that TLR3 deficiency in humans is associated with increased susceptibility to herpes simplex encephalitis, although this is mainly due to impairment of local responses rather than a defect a CD8 T cell responses [36]–[38].

The importance of IFN-I in controlling systemic HSV-1 infections has been confirmed in mice lacking IRF7 and STING, which have major defects in IFN-I responses [52], [53]. Our data and others [19] suggest that although pDC contribute to IFN-I production during systemic HSV-1, they are not the only source. While pDC are mainly important for early IFN-I secretion, TLR3-expressing cells, such as CD8α DC, are essential for IFN-I production at later time points. Other TLR3-expressing cells that might contribute to IFN-I during HSV-1 infection may include both hematopoietic and non-hematopoietic cells such as marginal zone macrophages, fibroblasts and stromal cells [36]–[38], [54]. Taken together, our study indicates that some cell types are more important than others for establishing antiviral immunity to a given virus and that cell types expressing different viral sensors promote IFN-I production in a time-dependent manner during systemic HSV infections.

## Materials and Methods

### Ethics statement

For mouse studies at Washington University, the principles of good laboratory animal care were carried out in strict accordance with the recommendations in the Guide for the Care and Use of Laboratory Animals of the National Institutes of Health and following the International Guiding Principles for Biomedical Research Involving Animals. The protocols were approved by the Animal Studies Committee at Washington University (#20100280).

### Mice and treatments

C57BL/6, CLEC4C-DTR Tg and TLR3^−/−^ mice were bred in house. Batf3^−/−^ mice were kindly provided by K. Murphy and W. KC (Washington University School of Medicine, St. Louis, MO). All mice were on a C57BL/6 background and used at 6–12 weeks of age. Diphtheria toxin (DT, Sigma-Aldrich) was injected i.p. into CLEC4C-DTR Tg mice (100–120 ng/mouse) 24 h prior to infection and every other day after. Seven days prior to vaginal infections, female CLEC4C-DTR Tg mice were injected subcutaneously with 2 mg of Depo-Provera or medroxyprogesterone acetate in PBS as previously described [55].

### Infections

HSV-1 KOS strain, provided by D. Leib (Dartmouth College, Hanover, NH), was injected i.v. at 1×10^7^ pfu or in the footpad at 1×10^5^ pfu. HSV-2 strain 186TKΔkpn was provided by A. Iwasaki (Yale University, New Haven, CT). Mice were infected with 5×10^6^ pfu i.v. or 1×10^3^ pfu ivag of HSV-2. For susceptibility studies, mice were infected with different doses of HSV-2 either i.v. or ivag (specified in figures/legends).

### Viral titers

Vaginal washes and vaginas and cervix from HSV-2-infected mice were removed on day 2 p.i. weighed and stored at −80°C in 1 ml of plain DMEM. Spleens and DLN from HSV-1-infected mice were removed on day 7 p.i. and stored at −80°C in 1 ml of plain DMEM. On the day of titering, samples were thawed in a 37°C water bath then homogenized with a Roche MagNA Lyser. Samples were centrifuged for 5 min at 5000 rpm then homogenates were serially diluted in plain DMEM. Dilutions were applied to Vero cell monolayers in 12 well plates for 1 h at 37°C. After the incubation period, an overlay containing complete DMEM and 1% methylcellulose was added and plates were incubated for 2–3 days. A second overlay with neutral red was applied 8 h before counting plaques.

### Cell preparations

Spleens and lymph nodes were minced and digested for 45 min at 37°C in RPMI 1640 with collagenase D (Sigma-Aldrich). Single cell suspensions were prepared by passage through nylon mesh cell strainers (BD Biosciences). Red blood cells were lysed with RBC lysis buffer (Sigma-Aldrich). Serum was separated from whole blood in serum collection tubes and stored at −20°C until analysis.

### Adoptive transfers

CD8 T cells from gBT-I Tg mice [33] were isolated by negative selection using the CD8α^+^ T cell Isolation Kit II according to the manufacturer instructions (Miltenyi Biotec). Cells were labeled with 5 µM CFSE and injected i.v. into CLEC4C-DTR Tg mice at 1×10^6^ cells per mouse. One day after transfer, mice were injected with PBS or DT. One day later, mice were infected with 1×10^7^ pfu HSV-1 i.v. Spleens were analyzed for CD8^+^CFSE^+^ T cells on day 3 p.i.

### Cell lines and tissue culture

EL4 cells were grown in complete RPMI: RPMI 1640 (Gibco/Invitrogen) with 10% bovine calf serum (BCS), 1% glutamax, 1% nonessential amino acids, 1% sodium pyruvate and 1% kanamycin sulfate (Gibco/Invitrogen). Vero cells were grown in DMEM with 10% BCS, 1% glutamax, 1% hepes and 1% pen/strep (Gibco/Invitrogen). Primary cells were cultured in complete RPMI with 10% fetal calf serum (FCS, Hyclone).

### Antibodies and flow cytometry

The following reagents were from BD Biosciences, eBioscience or Biolegend. Fluorochrome labeled anti-CD3 (145-2C11), anti-NK1.1 (PK136), anti-CD8α (53–6.7), anti-CD4 (MZ3), anti-IFN-γ (XMG1.2), CD69 (H1.2F3), CD107a (1D4B), CD25 (PC61), and Streptavidin. Fc receptors were blocked before surface staining with supernatant from HB-197 cells (ATCC). Propidium iodide was used to gate out dead cells. CD107a staining on NK cells was performed as previously described [56]. Briefly, splenocytes from control and infected mice were cultured for 1 h with FITC-conjugated CD107a. After the 1 h incubation, Brefeldin A and monensin were added to wells for an additional 4 h. Cells were stained with NK1.1. and CD3 then fixed with 2% paraformaldehyde. All flow cytometry was conducted on a dual laser FACSCalibur flow cytometer (BD Biosciences) and analyzed with FlowJo software (Tree Star, Inc.).

### Intracellular staining

Spleen or lymph node cells from HSV-1-infected mice were incubated in complete RPMI alone or with HSV gB peptide (Anaspec, 10 µg/ml) in the presence of Brefeldin A. After 6 h, cells were intracellularly stained for IFN-γ using the Cytofix/Cytoperm kit from BD Biosciences. IFN-γ-producing NK and NKT cells were also detected by intracellular staining using the Cytofix/Cytoperm kit.

### Cytotoxicity assays

For Ag-specific lysis assays, lymph nodes from HSV-1-infected mice were resuspended in complete RPMI and serially diluted. EL4 cells were pulsed or not with HSV gB peptide (10 ng/ml) and labeled with 1 mCi/ml ^51^Cr for 2 h then incubated with effector cells at 37°C for 4 h. ^51^Cr release in supernatants was measured with a γ-counter.

### Cytokine analysis

Serum IFN-α and proinflammatory cytokine levels were determined by ELISA (PBL Interferon Source) or Cytometric Bead Array (BD Biosciences), respectively. For measurements of IFN-α in tissue homogenates from HSV-2 infected mice, vaginas and cervix were removed and weighed on day 2 p.i. then stored at −80°C in tubes with silica beads and lysis buffer: PBS, PMSF, protease inhibitors, Triton X-100. Samples were thawed in a 37°C water bath then homogenized with a Roche MagNA Lyser. Samples were centrifuged for 5 min at 5000 rpm and supernatants were diluted and used for ELISA.

### Statistical analysis

The statistical significance of differences in mean values was analyzed with unpaired, two-tailed Student's *t*-test using GraphPad Prism software. *P* values less than 0.05 were considered statistically significant.

## References

[1] Fitzgerald-Bocarsly P, Dai J, Singh S (2008) Plasmacytoid dendritic cells and type I IFN: 50 years of convergent history. Cytokine Growth Factor Rev 19: 3–19. 18248767 10.1016/j.cytogfr.2007.10.006 PMC2277216

[2] Blasius AL, Beutler B (2010) Intracellular toll-like receptors. Immunity 32: 305–315. 20346772 10.1016/j.immuni.2010.03.012

[3] Trinchieri G (2010) Type I interferon: friend or foe? J Exp Med 207: 2053–2063. 20837696 10.1084/jem.20101664 PMC2947062

[4] Gilliet M, Cao W, Liu YJ (2008) Plasmacytoid dendritic cells: sensing nucleic acids in viral infection and autoimmune diseases. Nat Rev Immunol 8: 594–606. 18641647 10.1038/nri2358

[5] Reizis B, Bunin A, Ghosh HS, Lewis KL, Sisirak V (2011) Plasmacytoid Dendritic Cells: Recent Progress and Open Questions. Annu Rev Immunol 29: 163–183. 21219184 10.1146/annurev-immunol-031210-101345 PMC4160806

[6] Swiecki M, Colonna M (2010) Unraveling the functions of plasmacytoid dendritic cells during viral infections, autoimmunity, and tolerance. Immunol Rev 234: 142–162. 20193017 10.1111/j.0105-2896.2009.00881.x PMC3507434

[7] Cisse B, Caton ML, Lehner M, Maeda T, Scheu S, et al (2008) Transcription factor E2-2 is an essential and specific regulator of plasmacytoid dendritic cell development. Cell 135: 37–48. 18854153 10.1016/j.cell.2008.09.016 PMC2631034

[8] Cervantes-Barragan L, Lewis KL, Firner S, Thiel V, Hugues S, et al (2012) Plasmacytoid dendritic cells control T-cell response to chronic viral infection. Proc Natl Acad Sci U S A 109: 3012–3017. 22315415 10.1073/pnas.1117359109 PMC3286988

[9] Swiecki M, Gilfillan S, Vermi W, Wang Y, Colonna M (2010) Plasmacytoid dendritic cell ablation impacts early interferon responses and antiviral NK and CD8(+) T cell accrual. Immunity 33: 955–966. 21130004 10.1016/j.immuni.2010.11.020 PMC3588567

[10] Takagi H, Fukaya T, Eizumi K, Sato Y, Sato K, et al (2011) Plasmacytoid dendritic cells are crucial for the initiation of inflammation and T cell immunity in vivo. Immunity 35: 958–971. 22177923 10.1016/j.immuni.2011.10.014

[11] Dzionek A, Fuchs A, Schmidt P, Cremer S, Zysk M, et al (2000) BDCA-2, BDCA-3, and BDCA-4: three markers for distinct subsets of dendritic cells in human peripheral blood. J Immunol 165: 6037–6046. 11086035 10.4049/jimmunol.165.11.6037

[12] Dzionek A, Sohma Y, Nagafune J, Cella M, Colonna M, et al (2001) BDCA-2, a novel plasmacytoid dendritic cell-specific type II C-type lectin, mediates antigen capture and is a potent inhibitor of interferon alpha/beta induction. J Exp Med 194: 1823–1834. 11748283 10.1084/jem.194.12.1823 PMC2193584

[13] Blasius AL, Cella M, Maldonado J, Takai T, Colonna M (2006) Siglec-H is an IPC-specific receptor that modulates type I IFN secretion through DAP12. Blood 107: 2474–2476. 16293595 10.1182/blood-2005-09-3746 PMC1895736

[14] Zhang J, Raper A, Sugita N, Hingorani R, Salio M, et al (2006) Characterization of Siglec-H as a novel endocytic receptor expressed on murine plasmacytoid dendritic cell precursors. Blood 107: 3600–3608. 16397130 10.1182/blood-2005-09-3842

[15] Whitley RJ (2001) Herpes simplex viruses; Knipe D, Howley P, editors. Philadelphia: Lippincott Williams and Wilkins. 2461–2510 p.

[16] Chew T, Taylor KE, Mossman KL (2009) Innate and adaptive immune responses to herpes simplex virus. Viruses 1: 979–1002. 21994578 10.3390/v1030979 PMC3185534

[17] Lund J, Sato A, Akira S, Medzhitov R, Iwasaki A (2003) Toll-like receptor 9-mediated recognition of Herpes simplex virus-2 by plasmacytoid dendritic cells. J Exp Med 198: 513–520. 12900525 10.1084/jem.20030162 PMC2194085

[18] Krug A, Luker GD, Barchet W, Leib DA, Akira S, et al (2004) Herpes simplex virus type 1 activates murine natural interferon-producing cells through toll-like receptor 9. Blood 103: 1433–1437. 14563635 10.1182/blood-2003-08-2674

[19] Hochrein H, Schlatter B, O'Keeffe M, Wagner C, Schmitz F, et al (2004) Herpes simplex virus type-1 induces IFN-alpha production via Toll-like receptor 9-dependent and -independent pathways. Proc Natl Acad Sci U S A 101: 11416–11421. 15272082 10.1073/pnas.0403555101 PMC509215

[20] Rasmussen SB, Sorensen LN, Malmgaard L, Ank N, Baines JD, et al (2007) Type I interferon production during herpes simplex virus infection is controlled by cell-type-specific viral recognition through Toll-like receptor 9, the mitochondrial antiviral signaling protein pathway, and novel recognition systems. J Virol 81: 13315–13324. 17913820 10.1128/JVI.01167-07 PMC2168887

[21] Davey GM, Wojtasiak M, Proietto AI, Carbone FR, Heath WR, et al (2010) Cutting edge: priming of CD8 T cell immunity to herpes simplex virus type 1 requires cognate TLR3 expression in vivo. J Immunol 184: 2243–2246. 20124105 10.4049/jimmunol.0903013

[22] Donaghy H, Bosnjak L, Harman AN, Marsden V, Tyring SK, et al (2009) Role for plasmacytoid dendritic cells in the immune control of recurrent human herpes simplex virus infection. J Virol 83: 1952–1961. 19073735 10.1128/JVI.01578-08 PMC2643779

[23] Lund JM, Linehan MM, Iijima N, Iwasaki A (2006) Cutting Edge: Plasmacytoid dendritic cells provide innate immune protection against mucosal viral infection in situ. J Immunol 177: 7510–7514. 17114418 10.4049/jimmunol.177.11.7510

[24] Shen H, Iwasaki A (2006) A crucial role for plasmacytoid dendritic cells in antiviral protection by CpG ODN-based vaginal microbicide. J Clin Invest 116: 2237–2243. 16878177 10.1172/JCI28681 PMC1518794

[25] Asselin-Paturel C, Brizard G, Pin JJ, Briere F, Trinchieri G (2003) Mouse strain differences in plasmacytoid dendritic cell frequency and function revealed by a novel monoclonal antibody. J Immunol 171: 6466–6477. 14662846 10.4049/jimmunol.171.12.6466

[26] Krug A, French AR, Barchet W, Fischer JA, Dzionek A, et al (2004) TLR9-dependent recognition of MCMV by IPC and DC generates coordinated cytokine responses that activate antiviral NK cell function. Immunity 21: 107–119. 15345224 10.1016/j.immuni.2004.06.007

[27] Blasius AL, Giurisato E, Cella M, Schreiber RD, Shaw AS, et al (2006) Bone marrow stromal cell antigen 2 is a specific marker of type I IFN-producing cells in the naive mouse, but a promiscuous cell surface antigen following IFN stimulation. J Immunol 177: 3260–3265. 16920966 10.4049/jimmunol.177.5.3260

[28] Asselin-Paturel C, Boonstra A, Dalod M, Durand I, Yessaad N, et al (2001) Mouse type I IFN-producing cells are immature APCs with plasmacytoid morphology. Nat Immunol 2: 1144–1150. 11713464 10.1038/ni736

[29] Dalod M, Salazar-Mather TP, Malmgaard L, Lewis C, Asselin-Paturel C, et al (2002) Interferon alpha/beta and interleukin 12 responses to viral infections: pathways regulating dendritic cell cytokine expression in vivo. J Exp Med 195: 517–528. 11854364 10.1084/jem.20011672 PMC2193614

[30] Yoneyama H, Matsuno K, Toda E, Nishiwaki T, Matsuo N, et al (2005) Plasmacytoid DCs help lymph node DCs to induce anti-HSV CTLs. J Exp Med 202: 425–435. 16061729 10.1084/jem.20041961 PMC2213078

[31] Stout-Delgado HW, Yang X, Walker WE, Tesar BM, Goldstein DR (2008) Aging impairs IFN regulatory factor 7 up-regulation in plasmacytoid dendritic cells during TLR9 activation. J Immunol 181: 6747–6756. 18981092 10.4049/jimmunol.181.10.6747 PMC2605669

[32] Stout-Delgado HW, Du W, Shirali AC, Booth CJ, Goldstein DR (2009) Aging promotes neutrophil-induced mortality by augmenting IL-17 production during viral infection. Cell Host Microbe 6: 446–456. 19917499 10.1016/j.chom.2009.09.011 PMC2779161

[33] Mueller SN, Heath W, McLain JD, Carbone FR, Jones CM (2002) Characterization of two TCR transgenic mouse lines specific for herpes simplex virus. Immunol Cell Biol 80: 156–163. 11940116 10.1046/j.1440-1711.2002.01071.x

[34] Schlitzer A, Loschko J, Mair K, Vogelmann R, Henkel L, et al (2011) Identification of CCR9- murine plasmacytoid DC precursors with plasticity to differentiate into conventional DCs. Blood 117: 6562–6570. 21508410 10.1182/blood-2010-12-326678

[35] Satpathy AT, Kc W, Albring JC, Edelson BT, Kretzer NM, et al (2012) Zbtb46 expression distinguishes classical dendritic cells and their committed progenitors from other immune lineages. J Exp Med 209: 1135–1152. 22615127 10.1084/jem.20120030 PMC3371733

[36] Zhang SY, Jouanguy E, Ugolini S, Smahi A, Elain G, et al (2007) TLR3 deficiency in patients with herpes simplex encephalitis. Science 317: 1522–1527. 17872438 10.1126/science.1139522

[37] Casanova JL, Abel L, Quintana-Murci L (2011) Human TLRs and IL-1Rs in Host Defense: Natural Insights from Evolutionary, Epidemiological, and Clinical Genetics. Annu Rev Immunol 29: 447–491. 21219179 10.1146/annurev-immunol-030409-101335

[38] Guo Y, Audry M, Ciancanelli M, Alsina L, Azevedo J, et al (2011) Herpes simplex virus encephalitis in a patient with complete TLR3 deficiency: TLR3 is otherwise redundant in protective immunity. J Exp Med 208: 2083–2098. 21911422 10.1084/jem.20101568 PMC3182056

[39] Schulz O, Diebold SS, Chen M, Naslund TI, Nolte MA, et al (2005) Toll-like receptor 3 promotes cross-priming to virus-infected cells. Nature 433: 887–892. 15711573 10.1038/nature03326

[40] Belz GT, Smith CM, Eichner D, Shortman K, Karupiah G, et al (2004) Cutting edge: conventional CD8 alpha+ dendritic cells are generally involved in priming CTL immunity to viruses. J Immunol 172: 1996–2000. 14764661 10.4049/jimmunol.172.4.1996

[41] Hildner K, Edelson BT, Purtha WE, Diamond M, Matsushita H, et al (2008) Batf3 deficiency reveals a critical role for CD8alpha+ dendritic cells in cytotoxic T cell immunity. Science 322: 1097–1100. 19008445 10.1126/science.1164206 PMC2756611

[42] Tussiwand R, Lee WL, Murphy TL, Mashayekhi M, Wumesh KC, et al (2012) Compensatory dendritic cell development mediated by BATF-IRF interactions. Nature 490: 502–507. 22992524 10.1038/nature11531 PMC3482832

[43] Swiecki M, Colonna M (2010) Accumulation of plasmacytoid DC: Roles in disease pathogenesis and targets for immunotherapy. Eur J Immunol 40: 2094–2098. 20853492 10.1002/eji.201040602 PMC3732170

[44] Sozzani S, Vermi W, Del Prete A, Facchetti F (2010) Trafficking properties of plasmacytoid dendritic cells in health and disease. Trends Immunol 31: 270–277. 20579936 10.1016/j.it.2010.05.004

[45] Kumagai Y, Takeuchi O, Kato H, Kumar H, Matsui K, et al (2007) Alveolar macrophages are the primary interferon-alpha producer in pulmonary infection with RNA viruses. Immunity 27: 240–252. 17723216 10.1016/j.immuni.2007.07.013

[46] Iannacone M, Moseman EA, Tonti E, Bosurgi L, Junt T, et al (2010) Subcapsular sinus macrophages prevent CNS invasion on peripheral infection with a neurotropic virus. Nature 465: 1079–1083. 20577213 10.1038/nature09118 PMC2892812

[47] Walunas TL, Bruce DS, Dustin L, Loh DY, Bluestone JA (1995) Ly-6C is a marker of memory CD8+ T cells. J Immunol 155: 1873–1883. 7543536

[48] Wrammert J, Kallberg E, Agace WW, Leanderson T (2002) Ly6C expression differentiates plasma cells from other B cell subsets in mice. Eur J Immunol 32: 97–103. 11754008 10.1002/1521-4141(200201)32:1<97::AID-IMMU97>3.0.CO;2-Y

[49] Geissmann F, Jung S, Littman DR (2003) Blood monocytes consist of two principal subsets with distinct migratory properties. Immunity 19: 71–82. 12871640 10.1016/s1074-7613(03)00174-2

[50] Blasius AL, Arnold CN, Georgel P, Rutschmann S, Xia Y, et al (2010) Slc15a4, AP-3, and Hermansky-Pudlak syndrome proteins are required for Toll-like receptor signaling in plasmacytoid dendritic cells. Proc Natl Acad Sci U S A 107: 19973–19978. 21045126 10.1073/pnas.1014051107 PMC2993408

[51] Blasius AL, Krebs P, Sullivan BM, Oldstone MB, Popkin DL (2012) Slc15a4, a gene required for pDC sensing of TLR ligands, is required to control persistent viral infection. PLoS Pathog 8: e1002915. 23028315 10.1371/journal.ppat.1002915 PMC3441671

[52] Honda K, Yanai H, Negishi H, Asagiri M, Sato M, et al (2005) IRF-7 is the master regulator of type-I interferon-dependent immune responses. Nature 434: 772–777. 15800576 10.1038/nature03464

[53] Ishikawa H, Ma Z, Barber GN (2009) STING regulates intracellular DNA-mediated, type I interferon-dependent innate immunity. Nature 461: 788–792. 19776740 10.1038/nature08476 PMC4664154

[54] Eloranta ML, Alm GV (1999) Splenic marginal metallophilic macrophages and marginal zone macrophages are the major interferon-alpha/beta producers in mice upon intravenous challenge with herpes simplex virus. Scand J Immunol 49: 391–394. 10219764 10.1046/j.1365-3083.1999.00514.x

[55] Zhao X, Deak E, Soderberg K, Linehan M, Spezzano D, et al (2003) Vaginal submucosal dendritic cells, but not Langerhans cells, induce protective Th1 responses to herpes simplex virus-2. J Exp Med 197: 153–162. 12538655 10.1084/jem.20021109 PMC2193810

[56] Alter G, Malenfant JM, Altfeld M (2004) CD107a as a functional marker for the identification of natural killer cell activity. J Immunol Methods 294: 15–22. 15604012 10.1016/j.jim.2004.08.008

